# Identification of non-actionable mutations with prognostic and predictive value in patients with advanced or metastatic non-small cell lung cancer

**DOI:** 10.1007/s12094-023-03362-8

**Published:** 2024-01-06

**Authors:** Mariano Provencio-Pulla, Diego Pérez-Parente, Sara Olson, Haroon Hasan, Begoña Campos Balea, Delvys Rodríguez-Abreu, Marta López-Brea Piqueras, Navdeep Pal, Samantha Wilkinson, Esther Vilas, Pedro Ruiz-Gracia, Manuel Cobo-Dols

**Affiliations:** 1 Hospital Universitario Puerta de Hierro-Majadahonda, Madrid, Spain; 2 Lung Cancer Squad, Roche Farma SA, C. de La Ribera del Loira, 50, 28042 Madrid, Spain; 3 Product Development Data Sciences, Genentech Inc, San Francisco, CA USA; 4 Hospital Universitario Lucus Augusti, Lugo, Spain; 5 Hospital Universitario Insular de Gran Canaria, Las Palmas de Gran Canaria, Spain; 6 Hospital Marqués de Valdecilla, Santander, Spain; 7 Product Development, Roche, Welwyn Garden City, UK; 8 Hospital Medical Oncology Intercenter Unit, Regional and Virgen de la Victoria University Hospitals, IBIMA, Málaga, Spain

**Keywords:** NSCLC, Non-actionable mutations, Prognosis, Real-world, Survival

## Abstract

**Introduction:**

Lung cancer is one of the most prevalent cancers and the leading cause of cancer death. Advanced non-small cell lung cancer (aNSCLC) patients frequently harbor mutations that impact their survival outcomes. There are limited data regarding the prognostic and predictive significance of these mutations on survival outcomes in the real-world setting.

**Methods:**

This observational retrospective study analyzed de-identified electronic medical records from the Flatiron Health Clinico-Genomic and FoundationCore® databases to identify patients with aNSCLC who initiated first-line immune checkpoint inhibitors (ICI; alone or in combination) or chemotherapy under routine care between 2016 and 2021. The primary objectives were to assess the prevalence of non-actionable mutations and to determine their association with overall survival (OS). Real-world progression-free survival (rwPFS) and real-world response (rwR) were investigated as secondary exploratory outcomes.

**Results:**

Based on an assessment of 185 non-actionable mutations in 2999 patients, the most prevalent mutations were *TP53* (70%), *KRAS* (42%), *CDKN2A/B* (31%), and *STK11* (21%). *STK11*, *KEAP1,* and *CDKN2A/B* mutations were significantly associated with lower rwR, shorter rwPFS and OS. *KRAS* mutations were clinically associated with shorter rwPFS in CIT-treated patients. Subgroup analysis revealed that fast progressors were significantly more likely to harbor *STK11*, *KEAP1,* and *CDKN2A/B* mutations. Accordingly, long-term survivors (LTS) showed a significantly lower prevalence of these mutations.

**Conclusion:**

Our results provide evidence on the prognostic value of *STK11*, *KEAP1,* and *CDKN2A/B* mutations in patients with aNSCLC. Further research is required to better understand the implications of these findings on patient management and future trial design and treatment selection.

**Supplementary Information:**

The online version contains supplementary material available at 10.1007/s12094-023-03362-8.

## Introduction

Lung cancer is the second most diagnosed cancer worldwide and the leading cause of cancer death to date [1]. Based on histology, it can be classified into small cell lung cancer and non-small cell lung cancer (NSCLC), which approximately represent 25% and 85% of lung cancer diagnoses, respectively. NSCLC can be further classified into lung adenocarcinoma (45–60%), squamous cell carcinoma (20–25%), and neuroendocrine carcinoma (10–15%), which are treated using diverse therapeutic strategies. Recently, the development of new targeted therapies and the use of immunotherapy have increased 5-year overall survival (OS) rates in patients with NSCLC [2, 3]. However, it remains unclear why certain subgroups of patients either do not respond to treatment or present significantly different survival rates than others.

In NSCLC, multiple driver mutations responsible for the initiation and maintenance of the cancer have been described (i.e., *EGFR*, *ALK*, *ROS1*, *BRAF*). This, in turn, has prompted the development of targeted therapies against them [3, 4]. However, most NSCLC patients do not harbor known driver mutations, or they have mutations that are not actionable [5]. In these cases, their care relies on immunotherapy ± chemotherapy [6], but they frequently present with non-driver or non-actionable mutations that affect disease progression, response to treatment and survival [3, 4]. Non-actionable mutations in certain tumor suppressor genes have been described to predict survival or response to treatment [6–9]. For instance, mutations in *STK11*, *KEAP1*, and *CDKN2A/B* genes have been linked to shorter survival and resistance to immunotherapy [10]. Nevertheless, the real-world evidence on non-driver and non-actionable mutations in advanced NSCLC (aNSCLC) patients remains limited, largely due to the small sizes of patient populations involved in the studies [11, 12]. Further research on the non-driver and non-actionable mutations associated with efficacy outcomes could help identify aNSCLC populations with high unmet needs, thus, guiding the choice of the best treatments based on their mutational pattern.

Our primary objective was to identify the predictive and prognostic value of non-driver and non-actionable mutations in a large real-world cohort of aNSCLC patients undergoing first-line (1L) chemotherapy and/or immune checkpoint inhibitors (ICI). Moreover, this study aimed to identify specific mutational profiles that could predict a patient’s response to treatment. Secondary objectives included: determining the overall prevalence of non-driver and non-actionable mutations in the selected patient cohort and characterizing the mutation profiles of specific patient subgroups known to respond differently to treatment, such as fast progressors (real-world time to progression [rwTP] < 3 months), long-term survivors (LTS, rwTP > 12 months), women, and never-smokers.

## Methods

### Study design

This observational retrospective cohort study was conducted employing the Flatiron Health–Foundation Medicine Clinico-Genomic database (FH-FMI CGDB), which includes patients from a subset of the Flatiron Health network of ~ 280 US cancer clinics (approximately 800 care sites). Retrospective longitudinal clinical data were derived from electronic health records and comprise patient-level structured and unstructured data including: patient demographics, precise diagnosis details such as staging, histopathology and biomarkers, along with selected treatment and outcomes. The clinical data are further enriched by linkage to genomic data that are procured from Foundation Medicine’s Core® database, enabling a deeper understanding of the patients’ genomic profiles.

### Participants

All patients with aNSCLC who had initiated 1L ICI (alone or in combination with chemotherapy) or chemotherapy under routine clinical practice between January 1, 2016, and June 30, 2021, were selected. 2016 was set as the start of the study period to include the following immunotherapy approvals in the United States: pembrolizumab, nivolumab, ipilimumab, durvalumab and atezolizumab. Eligible patients were only those who received 1L ICI and/or chemotherapy, had next-generation sequencing (NGS) reports prior to the 1L treatment end date, had tissue sample only, non-driver or non-actionable mutations (*EGFR*, *ALK*, *ROS1*, *BRAF V600E*, *RET*, *METex14*, *NTRK3* were considered as driver mutations), recorded activity within 90 days of advanced diagnosis, and absence of multiple primary cancers. There was no requirement for informed consent or ethical review and approval.

### Study outcomes

The study aimed to estimate the prevalence of non-actionable mutations deemed clinically relevant by expert opinion.

Real-world overall survival (rwOS) for each patient was measured, defined as the duration from the index date (the start of the 1L treatment to the date of death).

Real-world progression-free survival (rwPFS) was defined as the period from the initiation of 1L therapy until the earliest recorded occurrence of any form of disease progression or death.

Real-world response rates (rwR) were determined by analyzing the numbers and percentages of patients who responded to treatment compared to the total population. RwR was considered as complete response upon clearance of all lesions and pathological nodes, while partial response was recorded for a decrease ≥ 30% of the sum of the maximum diameters.

The study also evaluated the association between non-actionable mutational patterns and the following patient subgroups of special interest: fast progressors, LTS, women, [13], and never-smokers [14]. The last two were selected based on previous results from meta-analyses on immunotherapy.

### Statistical analysis

A descriptive analysis of the sociodemographic and clinical characteristics of this population was performed. Cox regression models were used to calculate hazard ratios (HR) and 95% confidence intervals (CI) for rwPFS and rwOS. Logistic regression models were used to obtain odds ratios (OR) and 95% CI for rwR.

To balance the differences in baseline characteristics between the mutated and wild-type groups, inverse probability weights were utilized. Propensity score models were employed to determine the associations between mutational status and rwPFS, rwOS, and rwR. This involved the inclusion of various prognostic variables: age, Eastern Cooperative Oncology Group performance status (ECOG PS) at 1L therapy, race, sex, type of diagnosis, smoking status, time from diagnosis to 1L therapy start date, histology, and brain/CNS metastasis at baseline. For each covariate, the balance between mutated and wild-type groups was evaluated using standard mean difference (SMD), where ideal balance was defined as SMD < 0.1. Multivariable modelling was restricted to mutations where the exposure group had at least 10 patients. The statistical analysis was performed using R package version 4.1.0. The significance level was set to alpha 0.05.

## Results

### Characteristics of the study population

**Fig. 1:**
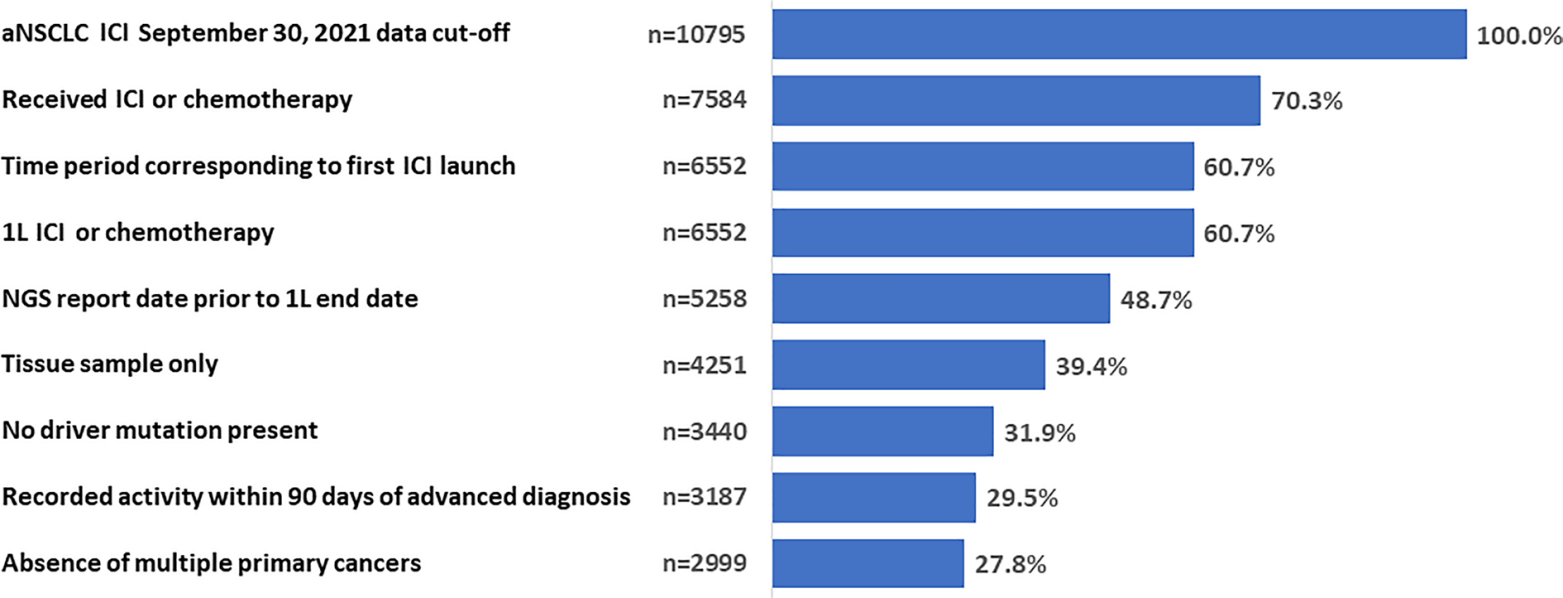
Cohort selection. *1L* first-line treatment, *aNSCLC* advanced or metastatic non-small cell lung cancer, *ICI* immune checkpoint inhibitors, *NGS* next-generation sequencing

A total of 10,795 patients with aNSCLC, who had initiated 1L ICI (alone or in combination with chemotherapy) or chemotherapy at data cut-off were selected. Of these, the 2999 patients (27.8%) who met the inclusion/exclusion criteria for this in-depth analysis were finally included in the study cohort (Fig. 1).

In the overall population, 65.3% received a ICI-containing treatment while the remaining 34.7% were treated with chemotherapy (Table 1). Mean age was 67.9 years. A higher proportion of patients aged ≥ 65 years was observed in the ICI-containing group compared to the chemotherapy one. Approximately half (53.4%) of the patients were men and nearly all had a history of smoking (95.1%). Histologically, non-squamous cell carcinoma was the most frequent type (67.7%), with a higher prevalence in the ICI-treated group. Overall, 58.4% of the patients had de novo diagnosis (63.2% in patients receiving ICI-containing treatment). Generally, ECOG PS was good (61.7% scoring 0). Regarding programmed cell death-ligand 1 (PD-L1) status, 32.1% patients in the ICI-containing group showed a PD-L1 expression of at least 50% (Table 1).

**Table 1:** Baseline characteristics of study patients

Variable	Categories	Overall (*N* = 2999)	Chemotherapy (*n* = 1042)	ICI-containing (*n* = 1957)
Gender, n (%)	Male	1600 (53.4)	559 (53.6)	1041 (53.2)
Age at advanced diagnosis, *n* (%)	Mean (SD)	67.87 (9.39)	67.36 (9.23)	68.15 (9.46)
	< 65 years	1058 (35.3)	408 (39.2)	650 (33.2)
	65–75	1116 (37.2)	364 (34.9)	752 (38.4)
	> 75	825 (27.5)	270 (25.9)	555 (28.4)
Histology, *n* (%)	Non-squamous cell carcinoma	2029 (67.7)	610 (58.5)	1419 (72.5)
	Squamous cell carcinoma	842 (28.1)	380 (36.5)	462 (23.6)
Smoking status, *n* (%)	History of smoking	2851 (95.1)	992 (95.2)	1859 (95.0)
	No or unknown	148 (4.9)	50 (4.8)	98 (5.0)
Advanced diagnosis, *n* (%)	De novo	1752 (58.4)	516 (49.5)	1236 (63.2)
	Recurrent	1247 (41.6)	526 (50.5)	721 (36.8)
ECOG PS, *n* (%)	0	687 (22.9)	237 (22.7)	450 (23.0)
	1	1150 (38.3)	420 (40.3)	730 (37.3)
	≥ 2	554 (18.5)	165 (15.9)	389 (19.8)
PD-L1 status, *n* (%)	High (≥ 50%)	817 (27.2)	189 (18.1)	628 (32.1)
	Low (1–49%)	758 (25.3)	250 (24.0)	508 (26.0)
	Negative (0%)	786 (26.2)	312 (3.9)	474 (24.2)
	Unknown	111 (3.7)	41 (3.9)	70 (3.6)

*ICI* immune checkpoint inhibitors, *ECOG PS* Eastern Cooperative Oncology Group performance status, *PD-L1* programmed cell death-ligand 1, *SD* standard deviation

### Prevalence of identified non-actionable mutations

**Fig. 2:**
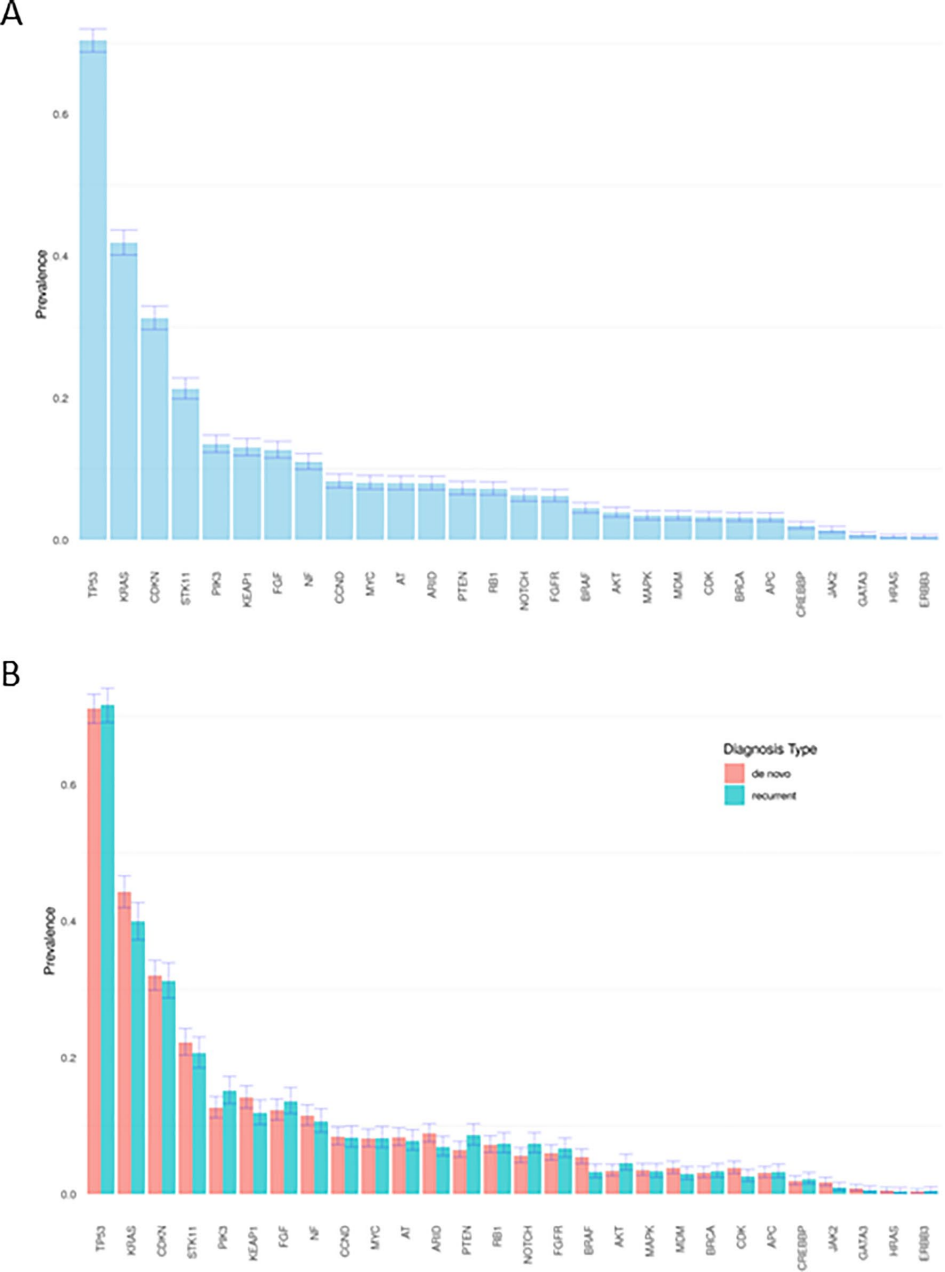
Prevalence of selected mutations in the overall population (**A**) and by advanced diagnosis type (**B**)

A total of 185 different non-actionable mutations were identified. These mutations were grouped into 58 mutation families. The most prevalent mutations in the overall population were *TP53*, observed in 70% of patients; *KRAS* in 42%; *CDKN2A/B* in 31%; and *STK11* in 21% (Fig. 2A). Interestingly, when the prevalence of these mutations was stratified by advanced diagnosis type (de novo or recurrent), no statistically significant differences were observed (Fig. 2B).

### Response and survival outcomes

**Fig. 3:**
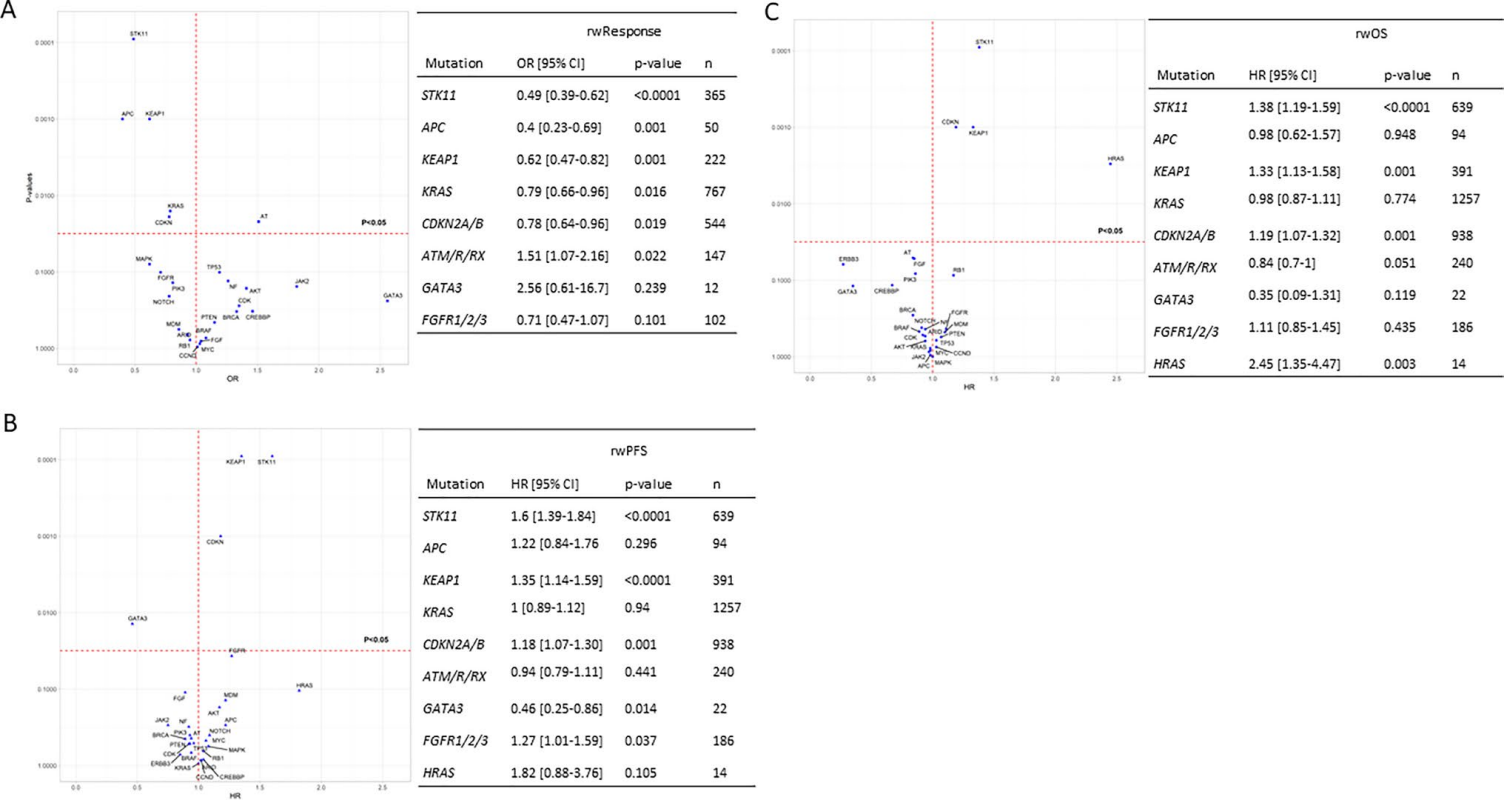
Volcano plots and real-world response values (**A**), real-world progression-free survival (**B**), and overall survival (**C**) in the overall population of patients with aNSCLC according to their mutational status. *CI* confidence interval, *HR* hazard ratio, *OR* odds ratio, *OS* overall survival, *PFS* progression-free survival, *rw* real-world

We assessed the association between the mutational status in patients with aNSCLC and the overall rwR, rwPFS, and rwOS. Of all identified mutations, *STK11*, *KEAP1,* and *CDKN2A/B* were significantly associated with all three effectiveness outcomes (Fig. 3). Patients harboring mutations in *STK11* showed a statistically significant lower rwR (OR: 0.49 [0.39–0.62], *p* < 0.0001), shorter rwPFS (OR: 1.38 [1.19–1.59], *p* < 0.0001) and reduced rwOS (OR: 1.6 [1.39–1.84], *p* < 0.0001) than the wild-type population. *APC* and *KRAS* mutations were only significantly associated with lower rwR (Fig. 3A), while *FGFR* and *HRAS* mutations were related to worse rwPFS and rwOS (Fig. 3B, C) respectively. In contrast, a significantly higher likelihood of response or rwPFS was associated with *ATM/R/RX* and *GATA3* mutations (Fig. 3B).

**Fig. 4:**
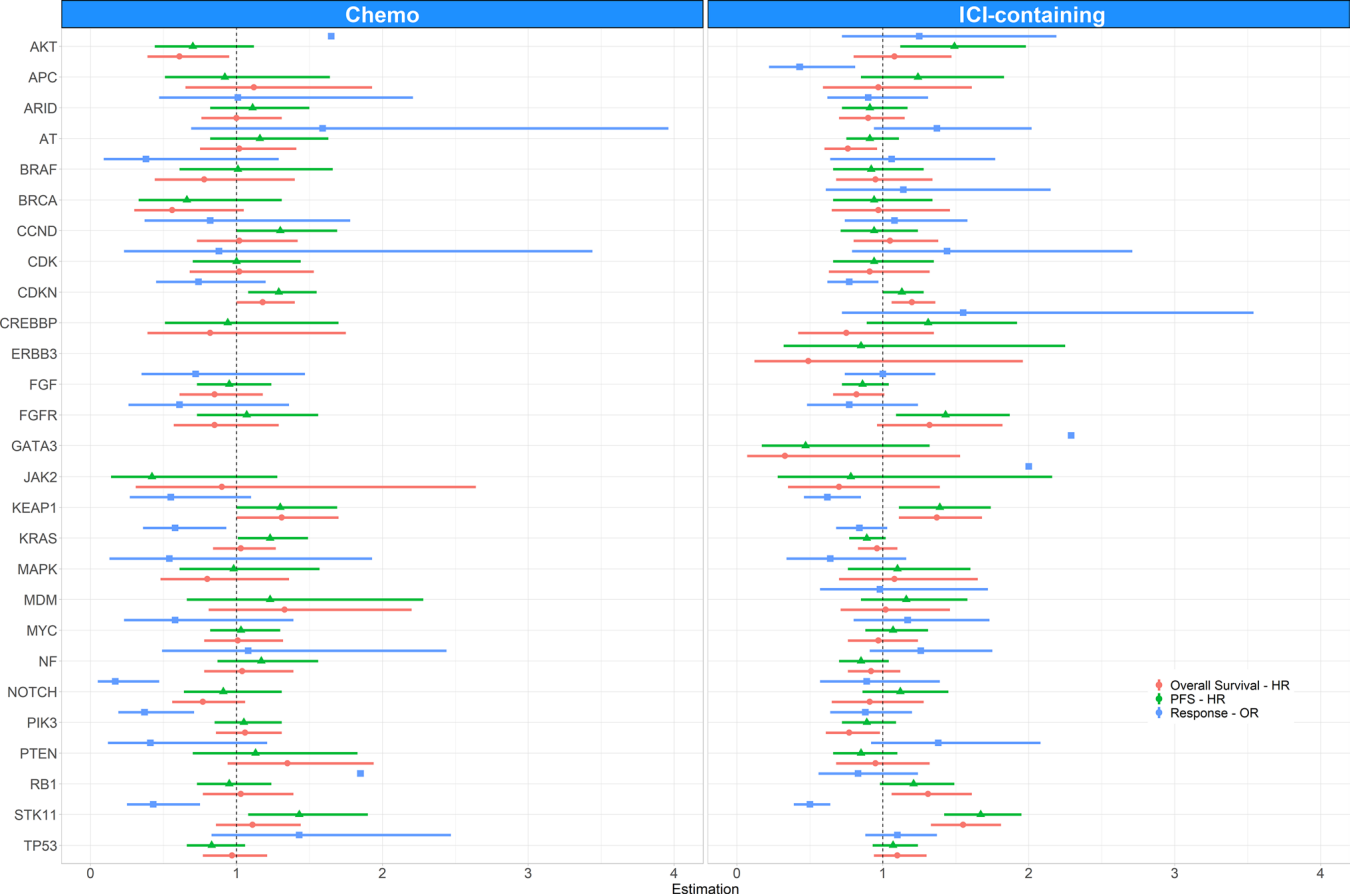
Forest plots of overall real-world response (blue), real-world progression-free survival (green), and overall survival (red) of the most relevant mutations by treatment group. *Chemo* chemotherapy, *ICI* immune checkpoint inhibitors, *HR* hazard ratio, *OR* odds ratio, *PFS* progression-free survival

Furthermore, we evaluated effectiveness outcomes depending on the treatment regimen (Fig. 4). Low rwR and short rwPFS were found in patients harboring *KRAS* mutations treated with chemotherapy. In the ICI-containing group, patients with *KEAP1* mutations showed low rwR and short rwPFS and rwOS, patients with *CDKN2A/B* mutations showed low rwR and short rwOS, while patients with *STK11* mutations showed short rwOS. Despite these results, we did not find statistically significant efficacy differences between treatment regimens in patients harboring *STK11*, *KEAP1* or *CDKN2A/B* mutations (Supplementary Fig. S1).

### Analysis of subgroups of especial interest

Fast progressors were characterized by a significantly higher prevalence of *STK11* (OR 1.68 [95% CI 1.41–2.01]), *KEAP1* (OR 1.60 [95% CI 1.29–1.99]), and *CDKN2A/B* (OR 1.28 [95% CI 1.09–1.50]) mutations compared with non-fast progressors. Consistent with these results, LTS showed a significantly lower prevalence of these mutations (Table 2). In the LTS subgroup, we also observed significantly lower prevalence of *FGFR1/2/3* mutations (OR 0.57 [95% CI 0.35–0.94]) and higher of *KRAS* mutations (OR 1.43 [95% CI 1.17–1.74]). In women, *APC* (OR 0.53 [95% CI 0.34–0.82]) and *FGFR1/2/3* (OR 0.58 [95% CI 0.43–0.79]) mutations were less frequent, while *KRAS* mutations were more frequent (OR 1.98 [95% CI 1.71–2.30]). In never-smokers, STK11 (OR 2.30 [95% CI 1.36–3.91]) and KEAP1 (OR 4.48 [95% CI 1.82–11.00]) mutations were significantly less prevalent. We also observed a trend towards a lower prevalence of FGFR1*/2/3* (OR 2.45 [95% CI 0.90–6.71]).

**Table 2:** Prevalence of selected non-actionable mutations in the overall study population and by treatment group

Mutation	All treatments combined			Chemotherapy			ICI-containing		
	OR [95% CI]	*p* value	mutated/wild-type	OR [95% CI]	*p* value	mutated/wild-type	OR [95% CI]	*p* value	mutated/wild-type
*STK11*									
Non-FP	1.68 [1.41–2.01]	< 0.001	294/1385	1.84 [1.36–2.48]	0.06	109/519	1.62 [1.30–2.01]	< 0.001	185/866
FP			338/945			112/290			226/655
Non-LTS	0.37 [0.27–0.50]	< 0.001	586/1919	0.42 [0.24–0.73]	0.002	206/689	0.34 [0.23–0.51]	< 0.001	380/1230
LTS			46/411			15/120			31/291
Male	1.06 [0.89–1.27]	0.48	333/1267	1.43 [10.6–1.93	0.02	103/456	0.91 [0.73–1.13]	0.40	230/811
Female			306/1093			118/365			188/728
Non-smoker	2.30 [1.36–3.91]	0.001	16/132	1.69 [0.75–3.81]	0.20	7/43	2.80 [1.40–5.86]	0.004	9/89
Smoker			623/2228			214/778			409/1450
*KEAP1*									
Non-FP	1.60 [1.29–1.99]	< 0.001	180/1499	1.59 [1.10–2.29]	0.0136	67/561	1.61 [1.23–2.10]	< 0.001	113/938
FP			207/1076			64/338			143/738
Non-LTS	0.61 [0.44–0.86]	0.004	346/2159	0.64 [0.34–1.19]	0.15	119/776	0.60 [0.40–0.91]	0.015	227/1383
LTS			41/416			12/123			29/293
Male	0.85 [0.69–1.06]	0.15	222/1378	1.05 [0.72–1.51]	0.81	69/490	0.77 [0.58–1.00]	0.05	153/888
Female			169/1230			62/421			107/809
Non-smoker	4.48 [1.82–11.00]	< 0.001	5/143	2.32 [0.71–7.57]	0.15	3/47	7.74 [1.90–31.57]	0.004	2/96
Smoker			386/2465			128/864			258/1601
*CDKN2A/B*									
Non-FP	1.28 [1.09–1.50]	0.002	482/1197	1.74 [1.34–2.28]	0.04	170/458	1.09 [0.90–1.33]	0.37	312/739
FP			436/847			158/244			278/603
Non-LTS	0.71 [0.57–0.89]	0.003	803/1702	0.60 [0.39–0.92]	0.02	297/598	0.77 [0.59–1.01]	0.06	506/1104
LTS			115/342			31/104			84/238
Male		0.05	525/1075	0.75 [0.58–0.98]	0.04	195/364	0.92 [0.76–1.11]	0.39	330/711
Female			413/986			139/344			274/642
Non-smoker		0.22	53/95	0.83 [0.46–1.50]	0.54	18/32	0.79 [0.52–1.21]	0.29	35/63
Smoker	0.81 [0.57–1.14]		885/1966			316/676			560/1290
*KRAS*									
Non-FP	1.05 [0.90–1.21]	0.53	694/985	1.10 [0.85–1.43]	0.46	214/414	0.98 [0.82–1.18]	0.87	480/571
FP			545/738			146/256			399/482
Non-LTS	1.43 [1.17–1.74]	< 0.001	1014/1491	1.15 [0.79–1.67]	0.46	309/586	1.51 [1.19–1.92]	< 0.001	705/905
LTS			225/232			51/84			174/148
Male	1.98 [1.71–2.30]	< 0.001	547/1053	1.82 [1.41–2.36]	0.004	160/399	2.09 [1.74–2.50]	< 0.001	387/654
Female			710/689			204/279			506/410
Non-smoker	1.20 [0.85–1.68]	0.30	56/92	1.27 [0.68–2.35]	0.45	15/35	1.18 [0.78–1.78]	0.44	41/57
Smoker			1201/1650			349/643			852/1007
*FGFR1/2/3*									
Non-FP	1.17 [0.87–1.58]	0.29	98/1581	1.01 [0.64–1.60]	0.96	51/577	1.39 [0.93–2.08]	0.10	47/1004
FP			87/1196			33/369			54/827
Non-LTS	0.57 [0.35–0.94]	0.03	167/2338	0.49 [0.21–1.14]	0.09	78/817	0.66 [0.36–1.22]	0.19	89/1521
LTS			18/439			6/129			12/310
Male	0.58 [0.43–0.79]	< 0.001	122/1478	0.49 [0.30–0.79]	0.003	58/501	0.66 [0.44–1.00]	0.05	64/977
Female			64/1335			26/457			38/878
Non-smoker	2.45 [0.90–6.71]	0.07	4/144	4.47 [0.61–32.82]	0.11	1/49	1.78 [0.55–5.72]	0.33	3/95
Smoker			182/2669			83/909			99/1760
*APC*									
Non-FP	1.11 [0.73–1.67]	0.63	51/1628	1.11 [0.59–2.10]	0.74	24/604	1.15 [0.67–1.99]	0.61	27/1024
FP			43/1240			17/385			26/855
Non-LTS	0.88 [0.48–1.59]	0.66	81/2424	0.51 [0.16–1.68]	0.26	38/857	1.17 [0.58–2.35]	0.66	43/1567
LTS			13/444			3/132			10/312
Male	0.53 [0.34–0.82]	0.003	64/1536	0.36 [0.17–0.74]	0.004	31/528	0.68 [0.39–1.20]	0.18	33/1008
Female			30/1369			10/473			20/896
Non-smoker	4.96 [0.69–35.81]	0.08	1/147	–	0.14	0/50	2.79 [0.38–20.41]	0.31	1/97
Smoker			93/2758			41/951			52/1807

*CI* confidence interval, *ICI* immune checkpoint inhibitors, *FP* fast progressors, *LTS* long-term survivors, *OR* odds ratio

In addition, in order to determine whether the treatment regimen was associated with fast progression or LTS in patients with a certain mutation, we performed a subgroup analysis. The logistic regression analysis did not show statistically significant differences between treatment groups. However, numerical differences were observed. Regarding *KEAP1*-mutated patients, there was a larger proportion of fast progressors in the ICI-containing-treated group (55.9%) compared to the chemotherapy-treated group (48.9%). We also observed a higher proportion of fast progressors among patients with *FGFR1/2/3* (53.5% vs 39.3%) and *APC mutations* (49.1% vs 41.5%) in the ICI-containing group. Lastly, *KRAS* (19.8% vs 14.2%) and APC-mutant patients (18.9 vs 7.3%) showed a larger proportion of LTS in the ICI-containing group compared with those who received chemotherapy.

## Discussion

To our knowledge, this is the largest real-world study to date describing the non-actionable mutational profile and its association with prognosis and predictive value in 1L patients with aNSCLC. In this dataset, we identified over 180 mutations, the most prevalent being *TP53*, *KRAS*, *CDKN2A/B,* and *STK11* mutations. These results are in line with those reported in the literature, except for a higher prevalence of *TP53* mutation, which is commonly associated with squamous histology [10, 15–17]. In the literature, there is very little evidence regarding *CDKN2A/B* in aNSCLC, with contradictory findings [10]. In this context, the large dataset used in our study and the significant outcomes confirms the importance of evaluating *CDKN2A/B* mutation as part of the mutational pattern in aNSCLC. We report that *STK11*, *KEAP1,* and *CDKN2A/B* mutations were significantly associated with poor prognosis in all effectiveness outcomes. In addition, *KRAS* mutations led to a lower rwR and clinically significant differences in rwPFS associated with different treatment regimens.

*STK11* is a tumor suppressor kinase, which negatively regulates the AMPK/mTOR pathway and is somatically inactivated in up to 30% of patients with NSCLC [18, 19]. A large real-world observational genome study found that *STK11* mutations had a negative prognostic value in patients with metastatic NSCLC treated with chemotherapy or immunotherapy [17]. Similarly, another observational study determined that *STK11* and *KEAP1* mutations were associated with shorter rwPFS and rwOS in all treatment groups, suggesting a prognostic but not predictive value for these biomarkers [16]. A more recent study showed that treatment with atezolizumab in patients harboring *STK11* or *KEAP1* mutations resulted in longer OS [20]. In our overall population, patients with mutated *STK11* showed lower rwR, rwPFS, and rwOS, and, in line with other reports, we did not observe statistically significant differences between chemotherapy and ICI-containing groups [16, 17].

*KEAP1* is a negative regulator of nuclear factor erythroid 2-related factor 2 involved in cell defense, and cytoprotective response to endogenous and exogenous stress [21]. Somatic mutations in *KEAP1* are found in about 20% of patients with NSCLC [19]. Goeman and colleagues showed that *KEAP1* mutations were associated with shorter survival outcomes in patients with aNSCLC [22]. Additional studies have also reported that patients harboring *KEAP1* mutations showed shorter survival regardless of treatment type [23, 24]. In this context, there are several ongoing clinical trials evaluating the efficacy of targeted therapy [25]. In our study, patients in the ICI-containing group exhibited worse outcomes compared to those treated with chemotherapy. This suggests a potential predictive role of *KEAP1* for ICI-containing treatment, although further studies are required to validate these results. Overall, harboring a *KEAP1* mutation led to lower rwR, rwPFS, and rwOS and our results are consistent with those published elsewhere [16, 20].

Mutations in *STK11* and *KEAP1* are associated with poor outcomes in patients with NSCLC, despite high TMB, including outcomes with PD-1 inhibitors [16, 26]. Inactivation of STK11 in lung cancer appears to result in an immunologically cold tumor microenvironment, with reduced T cell infiltration [26–28]. KEAP1 appears to interact functionally with STK11 [29] and these two proteins are significantly co-mutated in NSCLC, and result in a poor OS prognosis [30, 31]. OS and PFS outcomes in mSTK11 and mKEAP1 patients were improved by ICI treatment in several studies [32].

*CDKN2A/B* genes encode potent tumor suppressor proteins. In agreement, loss of function mutations in these genes negatively impact patient outcomes [33]. In this regard, our study showed that mutations in *CDKN2A/B* genes were associated with reduced rwR and shorter rwPFS and rwOS. Similarly, Gutiontov and colleagues showed that *CDKN2A* loss of function worsened clinical outcomes in aNSCLC patients treated with ICI [10]. In the same line, our ICI-containing group patients showed a trend towards lower rwR and shorter rwOS compared with those treated with chemotherapy. Only a few, small-scale studies have assessed the prevalence of *CDKN2A/B* mutations in aNSCLC and its impact on treatment outcomes [10, 34]; ours is one of the largest describing and confirming the role of *CDKN2A/B* in this setting.

*KRAS* is one of the most frequently mutated genes in cancer, being observed in up to 30% of patients with NSCLC [9, 35, 36]. Some studies suggest that *KRAS* mutations are associated with poor prognosis, while others found no correlation [35, 36]. In this context, a recent systematic review and meta-analysis analyzed 43 clinical studies to assess *KRAS* impact. Authors concluded that *KRAS* mutations may be associated with poor prognosis and response outcomes, but more evidence of its predictive value is needed [37]. We observed similar results, especially in patients treated with chemotherapy: *KRAS* mutation in these patients resulted in a numerically lower rwR and shorter rwPFS compared with patients in the ICI-containing group. Similarly, a recently published pooled analysis described that patients with *KRAS* mutations treated with ICI-containing therapy displayed a greater response and survival compared with those treated only with chemotherapy [38]. Overall, our data suggest that *KRAS* mutation may have predictive value for PFS in ICI-containing treated patients. However, additional research is needed to validate this clinical significance.

In the subgroup analyses, we observed that *STK11*, *KEAP1*, and *CDKN2A/B* mutations were significantly associated with fast disease progression, and together with *FGFR1/2/3* mutations with shorter survival. These results are consistent with our data on rwR, rwPFS, and rwOS; fast progressors seem to be more likely to harbor mutations in *STK11*, *KEAP1*, and *CDKN2A/B* and, therefore, have a poor prognosis. In a previous study, it was reported that *KEAP1* mutations are overrepresented in fast progressors, and it was suggested that they could define a molecular subset of patients characterized by resistance to chemotherapy [22]. Our results support this conclusion and also suggest that KEAP1 mutations could be a potential predictive biomarker for poor survival in patients treated with ICI-containing therapy. We observed a larger proportion of LTS in *KRAS*-mutated patients when treated with ICI-containing therapy, compared to chemotherapy. Given that this result is in line with the lower rwR and shorter rwPFS observed in patients treated with chemotherapy, it supports the idea of *KRAS* being a potential predictive biomarker.

The present study makes a significant contribution to the current literature on the mutational profile of patients with aNSCLC, although its retrospective nature could be considered a limitation. However, the real-world data and the large size of our dataset ensure representativity of the aNSCLC population. An additional limitation is the fact that certain mutations were observed in a limited number of patients, leading to CIs too large to draw any conclusions when carrying out comparisons. Furthermore, co-occurrence of mutations was not considered and the effect on tumor mutation burden was not investigated, making it impossible to exclude a potential selection bias. Finally, since sotorasib was approved while the study was ongoing (June 2021), we could not indicate whether patients with *KRAS* mutations were treated with this therapy, and we did not investigate different *KRAS* variants separately.

In conclusion, our study describes the prevalence and mutational pattern of 1L aNSCLC and shows that mutations in genes such as *STK11*, *KEAP1* and *CDKN2A/B* are significantly associated with poor efficacy outcomes. Thus, they could be considered prognostic factors. The same mutational profile was observed in de novo and recurrent patients, but other subgroups of patients, such as fast progressors and LTS, were characterized by distinct patterns of *STK11*, *KEAP1,* and *CDKN2A/B* mutations that could guide clinical decision making and help predict treatment response in patients with aNSCLC. Overall, our results contribute to the identification of novel biomarkers that could help clinicians determine the degree of treatment response expected from certain subgroups of patients. Further studies are needed to support these results and to evaluate their impact in clinical trial design or clinical decision making.

### Supplementary Information

Below is the link to the electronic supplementary material.

Supplementary file1 (PDF 152 KB)

## Data Availability

Qualified researchers may request access to individual patient-level data through the clinical study data request platform (https://vivli.org/). Further details on Roche’s criteria for eligible studies are available here: https://vivli.org/members/ourmembers/. For further details on Roche’s Global Policy on the Sharing of Clinical Information and how to request access to related clinical study documents, see here: https://www.roche.com/research_and_development/who_we_are_how_we_work/clinical_trials/our_commitment_to_data_sharing.htm.
