# Supplementary material for: Identification of non-actionable mutations with prognostic and predictive value in patients with advanced or metastatic non-small cell lung cancer
Source: Clin Transl Oncol. 2024 Jan 6;26(6):1384–94. doi: 10.1007/s12094-023-03362-8 (PMC11108921; doi:10.1007/s12094-023-03362-8)

Supplementary Figure 1. Forest plots comparing overall survival (A) and progression-free survival (B) in patients harboring *STK11*, *KEAP1* or *CDKN2A/B* mutations according to treatment regimen (CIT or chemotherapy). CIT: cancer immunotherapy; HR: hazard ratio.

A

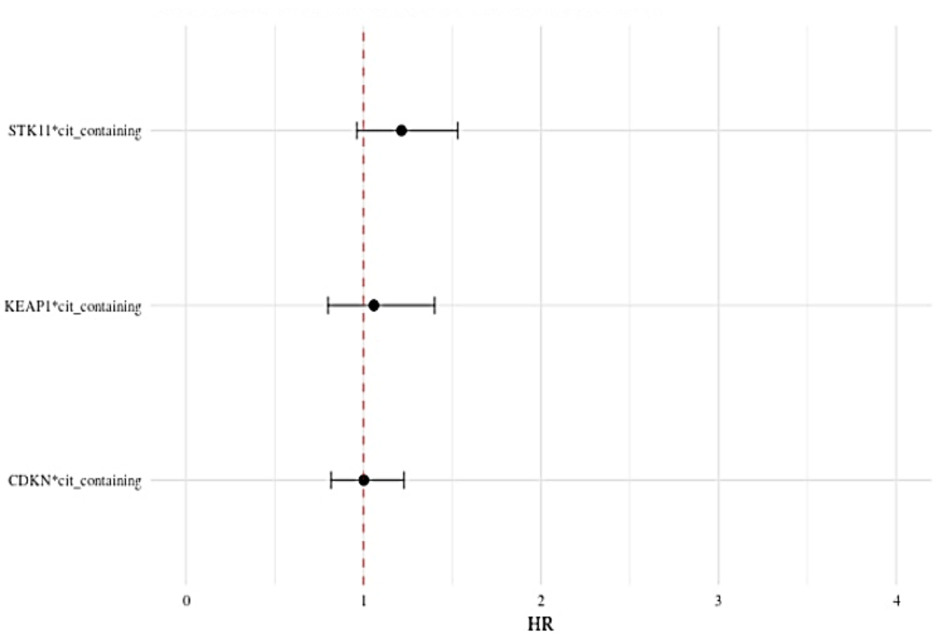

B

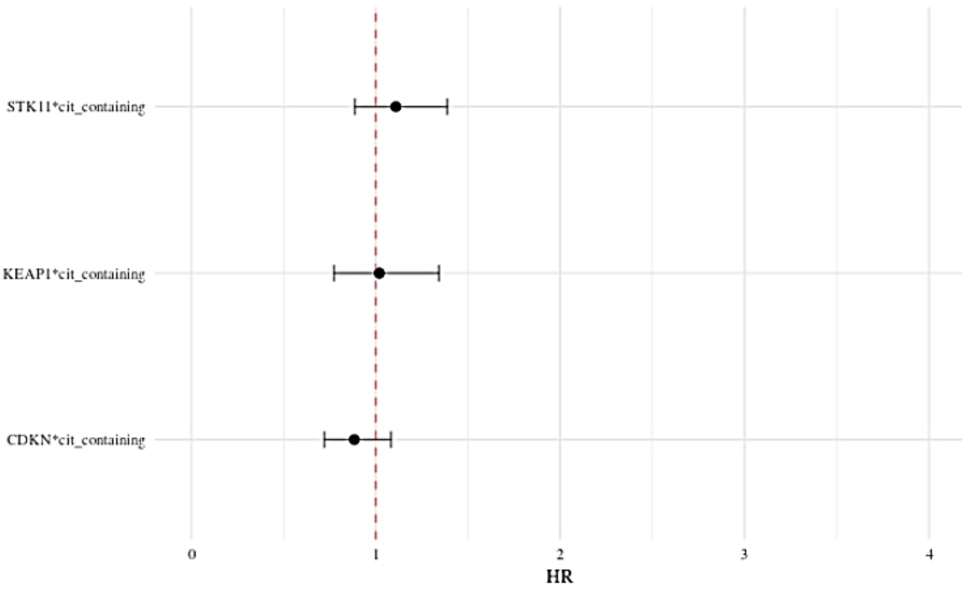

Supplement: Supplementary file 1 — Supplementary file1 (PDF 152 KB) [file 12094_2023_3362_MOESM1_ESM.pdf]
